# Cabozantinib can block growth of neuroendocrine prostate cancer patient-derived xenografts by disrupting tumor vasculature

**DOI:** 10.1371/journal.pone.0245602

**Published:** 2021-01-20

**Authors:** Mark P. Labrecque, Lisha G. Brown, Ilsa M. Coleman, Holly M. Nguyen, Daniel W. Lin, Eva Corey, Peter S. Nelson, Colm Morrissey

**Affiliations:** 1 Department of Urology, University of Washington School of Medicine, Seattle, Washington, United States of America; 2 Division of Human Biology, Fred Hutchinson Cancer Research Center, Seattle, Washington, United States of America; 3 Division of Public Health Sciences, Fred Hutchinson Cancer Research Center, Seattle, Washington, United States of America; 4 Department of Medicine, University of Washington School of Medicine, Seattle, Washington, United States of America; Columbia University, UNITED STATES

## Abstract

With the advent of potent second-line anti-androgen therapy, we and others have observed an increased incidence of androgen receptor (AR)-null small cell or neuroendocrine prostate cancer (SCNPC) in metastatic castration-resistant prostate cancer (mCRPC). Our study was designed to determine the effect of cabozantinib, a multi-targeted tyrosine kinase inhibitor that inhibits VEGFR2, MET and RET on SCNPC. Transcriptome analysis of the University of Washington rapid autopsy and SU2C mCRPC datasets revealed upregulated *MET* and *RET* expression in SCNPCs relative to adenocarcinomas. Additionally, increased *MET* expression correlated with attenuated AR expression and activity. *In vitro* treatment of SCNPC patient-derived xenograft (PDX) cells with the MET inhibitor AMG-337 had no impact on cell viability in LuCaP 93 (MET+/RET+) and LuCaP 173.1 (MET-/RET-), whereas cabozantinib decreased cell viability of LuCaP 93, but not LuCaP 173.1. Notably, MET+/RET+ LuCaP 93 and MET-/RET- LuCaP 173.1 tumor volumes were significantly decreased with cabozantinib treatment *in vivo*, and this activity was independent of MET or RET expression in LuCaP 173.1. Tissue analysis indicated that cabozantinib did not inhibit tumor cell proliferation (Ki67), but significantly decreased microvessel density (CD31) and increased hypoxic stress and glycolysis (HK2) in LuCaP 93 and LuCaP 173.1 tumors. RNA-Seq and gene set enrichment analysis revealed that hypoxia and glycolysis pathways were increased in cabozantinib-treated tumors relative to control tumors. Our data suggest that the most likely mechanism of cabozantinib-mediated tumor growth suppression in SCNPC PDX models is through disruption of the tumor vasculature. Thus, cabozantinib may represent a potential therapy for patients with metastatic disease in tumor phenotypes that have a significant dependence on the tumor vasculature for survival and proliferation.

## Introduction

The incidence of small cell or neuroendocrine prostate cancer (SCNPC) is on the rise in treatment-resistant castration-resistant prostate cancer (CRPC) [1–3]. Hallmarks of SCNPC include the loss of androgen receptor (AR) expression and upregulation of neuroendocrine (NE) programs that drive tumor progression [1,2]. Despite concerted efforts to understand the biological pathways responsible for treatment-induced transition of AR-expressing CRPC to AR-null SCNPC, there are still no approved targeted therapies for patients with SCNPC. Notably, AR-loss or attenuated AR signaling is associated with increased expression of the MET oncogene in CRPC, and MET has been suggested as a therapeutic target in AR therapy-resistant and AR-null CRPC [4]. Moreover, the RET proto-oncogene is also expressed in CRPC and preclinical studies targeting RET kinase activity in SCNPC have demonstrated reduced tumor growth [5,6].

Cabozantinib, a multi-kinase inhibitor of VEGFR2, MET, RET, and other kinases, has been assessed in clinical trials in CRPC patients. A Phase II trial indicated that cabozantinib had clinical activity, and the authors suggested that there was a potential cooperative role for MET and VEGF signaling in CRPC progression [7]. A Phase III clinical trial in men with progressive mCRPC after docetaxel and abiraterone and/or enzalutamide (COMET-1), revealed no significant differences in overall survival (OS) between cabozantinib (n = 682) and prednisone (n = 346) therapies [8]. In addition, COMET-2, a companion trial in the same heavily pre-treated mCRPC patient population, assessed cabozantinib versus mitoxantrone-prednisone and did not meet the primary endpoint of improved pain response [9]. While the failure of COMET-1 resulted in early termination of COMET-2, a retrospective analysis combining patients from both studies determined that cabozantinib was associated with improved OS after adjusting for prognostic factors [10]. Further, the authors suggested that a rationally selected patient population based on molecular biomarkers, such as *MET* expression, may benefit from cabozantinib treatment and warrants further investigation [10].

This study was designed to determine whether increased MET and RET expression and activity in SCNPC could be blocked by cabozantinib to inhibit tumor growth. Cell lines and Lucas Foundation Cancer of the Prostate (LuCaP) CRPC patient-derived xenograft (PDX) models were tested for responses to cabozantinib [11]. Using *in vitro* assays, we found that cabozantinib repressed the growth of cells from the SCNPC PDX model LuCaP 93 (MET+/RET+), whereas cells from the SCNPC PDX model LuCaP 173.1 (MET-/RET-) were resistant. Unexpectedly, *in vivo* treatment of both LuCaP 93 and 173.1 with cabozantinib significantly reduced tumor volume and increased survival compared to control animals. In addition, molecular analysis of treatment-resistant tumor cells remaining after cabozantinib treatment suggested that microvessel density was decreased in both PDX models and transcriptome analysis identified hypoxia and glycolysis pathways as the most significantly altered pathways in cabozantinib-treated tumors. These results indicate that inhibiting tumor vascularization and not necessarily MET or RET activity in tumor cells is the primary mechanism of cabozantinib-mediated tumor growth inhibition in SCNPC.

## Materials and methods

### Cell lines and tumor models

NCI-H660 cells (obtained directly from ATCC, CRL-5813) were maintained in HITES media (ATCC) with 5% fetal bovine serum (Atlanta Biologicals) in humidified Steri-Cult CO_2_ incubators (Thermo Scientific). LuCaP patient-derived xenograft tumors (established in-house; [12]) were harvested and dissociated using the human Tumor Dissociation Kit (Miltenyi Biotec) according to manufacturer’s protocol.

### Viability assay

LuCaP PDX cells (2.0X10^4^) and NCI-H660 cells (5.0x10^3^) were seeded in 96-well plates in either phenol red-free RPMI 1640 media (Gibco) supplemented with 5% charcoal/dextran treated fetal bovine serum (Atlanta Biologicals) and 1X pen/strep (Gibco) or HITES media (ATCC). Cells were treated 4–6 hours after seeding with vehicle, AMG-337 (50 nM) or cabozantinib (2.5 μM) in three replicate wells. Viability was assessed at time 0 and 72 hours post treatment using the CellTitre Glo 3D-Cell Viability Assay (Promega) according to manufacturer’s protocols.

### In vivo testing

All procedures used in this study were approved by the Institutional Animal Care and Use Committee at the University of Washington (Protocol Number: 3202–01) and the Animal Care and Use Review Office (ACURO) in accordance with the National Institutes of Health and US Department of Defense guidelines. SCNPC patient-derived xenograft models LuCaP 93 and LuCaP 173.1 were derived from a transurethral resection of the prostate and a liver metastasis respectively [2,12]. The LuCaP PDX models were maintained by serial passaging in CB-17, severe combined immunodeficient (SCID) male mice as described previously [12]. CB-17 SCID mice (Charles River Laboratories) were implanted subcutaneously with either LuCaP 93 or 173.1 tumor tissue. Animals underwent rolling enrollment once tumors exceeded 100 mm^3^ and were randomized into two groups. Group 1 received control vehicle, by oral gavage, 200 μL, 4 times weekly for up to 7.5 weeks. Group 2 received 30 mg/kg cabozantinib in vehicle, by oral gavage, 100 μL, 5 times weekly for up to 7.5 weeks. Three mice per group were euthanized at 5 days post enrollment for early time point analysis, and the remaining 14 mice per group were dosed for up to 7.5 weeks. Tumor volumes (TV) were measured using Fowler Ultra-Cal calipers (calculated as LxHxWx0.5236). Tumor volumes and body weights were collected twice weekly. In addition, animals were monitored at least 3 times weekly for health conditions and abnormal behaviors associated with pain and distress. Animals were euthanized after the 7.5 week dosing period, if tumor volumes exceeded 1,000 mm^3^, if body weight fell below 20%, or if animals showed other signs of health compromise (i.e. body condition score < 2, ulcerating tumors, lethargy, piloerection). All efforts were made to minimize suffering and no unexpected mortality occurred outside of planned euthanasia or humane endpoints. For euthanasia, mice were anesthetized by an intraperitoneal injection of a ketamine/xylazine (130 mg/8.8 mg/kg) cocktail and bled by cardiac puncture, which was immediately followed by cervical dislocation. At sacrifice, the tumors were divided equally into paraffin blocks and flash frozen for subsequent molecular analyses.

### RNA sequencing

RNA was isolated from flash frozen tissue from 6 LuCaP 93 tumors (3 treated with vehicle and 3 treated with cabozantinib for 5 days) using RNA STAT-60 (Tel-Test). For the LuCaP 173.1 study, all cabozantinib-treated animals had insufficient viable tumor for analysis. The isolated RNA was then purified with Qiagen RNeasy Kit (Qiagen Inc.) according to the manufacturer’s protocol utilizing the optional DNase treatment in solution prior to purification. RNA concentration, purity, and integrity were assessed by NanoDrop 2000 (Thermo Fisher Scientific Inc) and 2100 Bioanalyzer (Agilent Technologies). RNA-Seq libraries were constructed from 1 μg total RNA using the Illumina TruSeq Stranded mRNA LT Sample Prep Kit according to the manufacturer’s protocol. Barcoded libraries were pooled and sequenced on a NovaSeq S1 100 flowcell generating 50 bp paired end reads. Sequencing reads were mapped to the hg38 human and mm10 mouse genomes using STAR.v2.7.3a. All subsequent analyses were performed in R. Sequences aligning to the mouse genome deriving from potential contamination with mouse tissue were removed from the analysis using XenofiltR [13]. Gene level abundance was quantitated from the filtered human alignments using Genomic Alignments [14]. Differential expression was assessed using transcript abundances as inputs to the limma [15], filtered for a minimum expression level of at least 1 count per million reads (CPM) in three samples prior to testing, using the Benjamin-Hochberg false discovery rate (FDR) adjustment.

Transcriptome analysis of UW rapid autopsy and SU2C mCRPC specimens and LuCaP PDX models was conducted using previously published RNA-Seq datasets [2,16]. RNA sequencing data for the UW rapid autopsy and LuCaP PDX biospecimens can be retrieved at Gene Expression Omnibus (GEO) through accession number GSE126078. At the time of this report, the most recent SU2C mCRPC sequencing data can be accessed through DbGaP at phs000915.v2.p2. RNA sequencing data generated in this report can be retrieved at GEO through accession number GSE148538. The molecular phenotypes of mCRPC biospecimens from UW and SU2C mCRPC cohorts and LuCaP PDX models were determined previously [2]. Briefly, mCRPC transcriptomes were stratified using AR and NE signatures and then molecular profiles were validated through immunohistochemistry and/or cluster analysis [2].

### Pathway analysis

Gene expression results were ranked by their limma statistics and used to conduct Gene Set Enrichment Analysis (GSEA) to determine patterns of pathway activity in treatment groups utilizing the pathways from within the MSigDBv7 [17]. Single sample enrichment scores were calculated using GSVA with default parameters using genome-wide log_2_ FPKM values as input, and 10-gene neuroendocrine (NE) and androgen-regulated (AR) signatures from Bluemn et al. [18,19].

### Tissue microarray construction

Each tumor was fixed in buffered formalin and embedded in paraffin. A tissue microarray (TMA) was constructed using duplicate 1 mm diameter cores from control and cabozantinib-treated tumors. LuCaP 93 and 173.1 PDX tissue microarrays were constructed from 56 control and treated tumors (30 control; 26 treated).

### Immunohistochemistry

Five-micron sections of the TMAs were deparaffinized and rehydrated in sequential xylene and graded ethanol. Antigen retrieval was performed in 10 mM citrate buffer (pH 6.0) in a pressure cooker for 30 minutes. Endogenous peroxidase and avidin/biotin were blocked respectively (Vector Laboratories Inc.). Sections were then blocked with 5% normal goat-horse-chicken serum, incubated with primary antibody (Ki67 Dako M7240; 1:100, CD31 Abcam ab124432; 1:200, Hexokinase II Abcam ab1048363; 1:250), incubated with biotinylated secondary antibody (Vector Laboratories Inc.), followed by ABC reagent (Vector Laboratories Inc.), and stable DAB (Invitrogen Corp.). All sections were lightly counterstained with hematoxylin and mounted with Cytoseal XYL (Richard Allan Scientific). Mouse MOPC-1 or rabbit IgG were used as negative controls as appropriate. Ki67 and microvessel density (CD31) scores were determined as described previously [20]. To assess cell proliferation, positively and negatively staining tumor cell nuclei were counted in five fields at 200X magnification. To assess microvessel density after scanning at low magnification for microvessel hotspots, blood vessels were counted in three representative fields of each tissue section at 200× magnification. A blood vessel was defined as any CD31 immunostained endothelial cell cluster separated from adjacent vessels. Unusable samples, including missing, or necrotic tissue cores, were excluded from final analysis.

### Immunoblot analysis

Whole cell protein extracts from LuCaP PDX models and cell lines were obtained using the Nuclear Extract Kit (Active Motif) according to manufacturer’s protocols. Quantification of total protein was determined using the RC DC Protein Assay (Bio-Rad) according to manufacturer’s protocols. Twenty to thirty micrograms of total protein lysate were electrophoresed on 4–15% Bis-Tris gels (Bio-Rad) with 1x Tris/Glycine/SDS Buffer (Bio-Rad). The proteins were transferred to nitrocellulose that was blocked with 5% Blotting-Grade Blocker (Bio-Rad) in TBS/0.1% Tween-20 and subsequently probed with primary and secondary antibodies (c-MET Cell Signaling 8198; 1:1000, RET AbCAM ab134100; 1:750, VEGFR2 Cell Signaling 9698; 1:1000, and ACTIN Sigma A2228; 1:2500). Proteins were visualized using Clarity Western ECL Substrate (Bio-Rad).

### Statistical analysis

Tumor volume (TV), body weight (BW), and overall survival (OS) between control and treated animals were compared when >2 animals remained in each group. Differences in TV and BW between control and treated animals were calculated using unpaired t-tests with significance set at p ≤ 0.05. Kaplan-Meier analysis was performed for overall survival using the log-rank (Mantel-Cox) test. For IHC comparison, unpaired t-tests with unequal variances and significance set at p ≤ 0.05 were utilized. Boxplots of signature scores were compared using the ggpubr (https://CRAN.R-project.org/package=ggpubr) stat_compare_means function by unpaired student’s t-test using equal variances and controlled for multiple testing using the Holm method. Statistical significance cutoffs are listed in figure legends. Boxplots of log_2_ FPKM values were compared using a 1-way ANOVA with Tukey’s multiple comparisons test in Prism (GraphPad). Pearson's correlation coefficient was used to study the relationships between variables shown in scatterplots using the cor.test function in R.

## Results

### MET and RET are upregulated in metastatic SCNPC biospecimens

**Fig 1:**
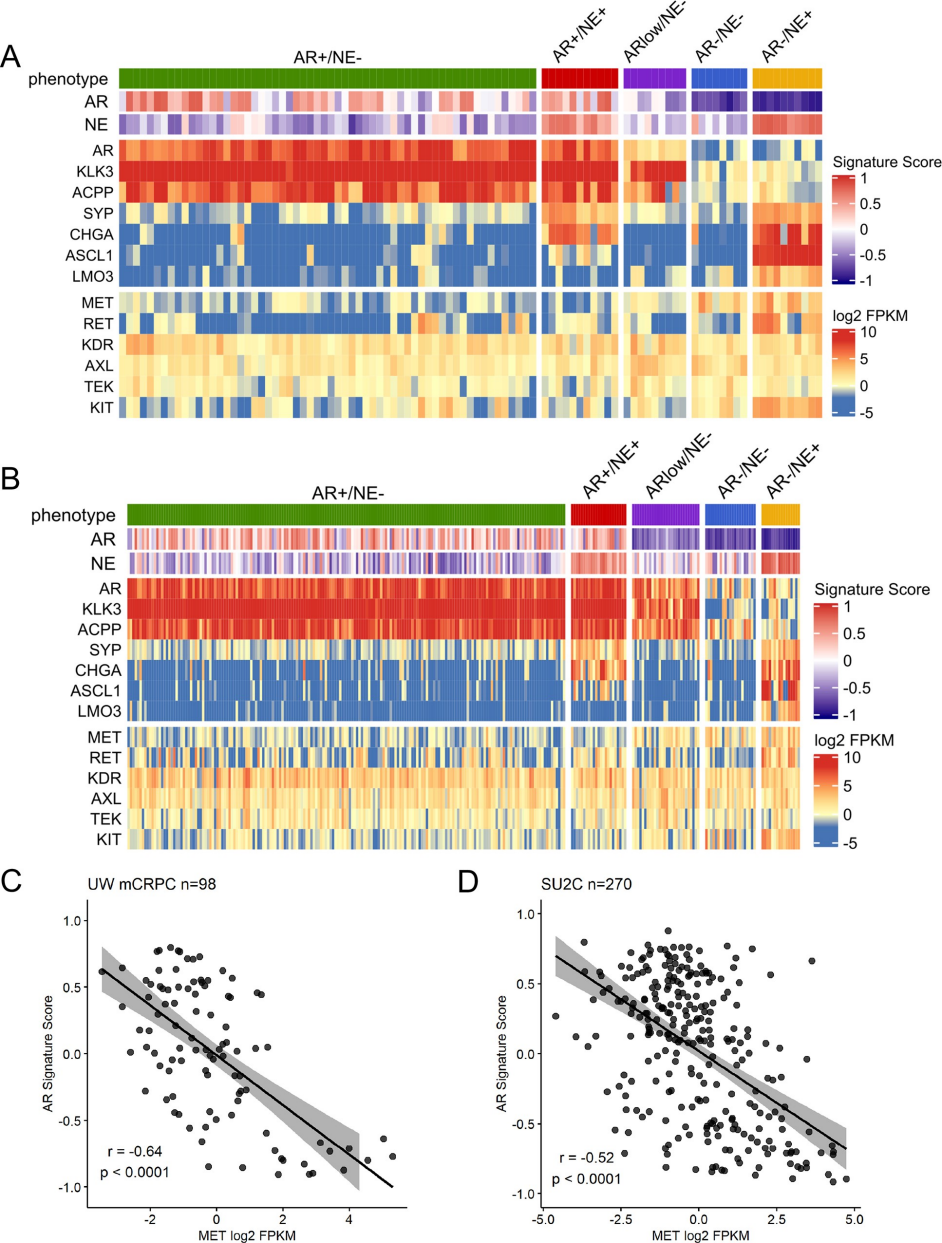
Metastatic SCNPC upregulates *MET* and *RET*. Whole transcriptome RNA-Seq heatmaps of (A) UW rapid autopsy cohort (n = 98) and (B) SU2C cohort (n = 270) depicting AR-associated genes (*AR*, *KLK3* and *ACPP*), NE-associated genes (*SYP*, *CHGA*, *ASCL1* and *LMO3*), targets of cabozantinib (*MET*, *RET*, *VEGFR2/KDR*, *AXL*, *TEK*, *KIT*), and AR and NE activity scores. Results are expressed as log_2_ FPKM (transcripts) or as GSVA scores (activities) and are colored according to scale. Correlation analysis of AR activity scores (GSVA) and *MET* transcript expression (log_2_ FPKM) in (C) UW and (D) SU2C cohorts.

The loss of AR expression or AR signaling has been associated with upregulated MET expression in CRPC [4] but the expression of MET in distinct mCRPC molecular phenotypes has not been determined. Using 98 mCRPC tumor specimens from the University of Washington rapid autopsy (UW) cohort [2] and 270 tumor specimens from the SU2C cohort [16], we confirmed through RNA-Seq that *MET* transcript expression is significantly upregulated in SCNPC tumors (AR-/NE+; p<0.0001) compared to AR-high metastases (AR+/NE-; **Figs 1A, 1B and S1**). The molecular phenotypes of mCRPC biospecimens represented in Fig 1 were determined previously using established AR and NE transcriptional signatures [2]. Notably, *MET* expression was moderately negatively correlated with AR activity score in both the UW (Pearson’s correlation r = -0.64; p<0.0001) and SU2C (Pearson’s correlation r = -0.52; p<0.0001) datasets (**Fig 1C and 1D**). To assess MET pathway activity in SCNPC metastases, we conducted gene set enrichment analyses (GSEA) using C2 curated gene sets from the chemical and genetic perturbations (CGP) and canonical pathways (CP) in MSigDB. Of the 17 MET-associated gene sets from C2-CGP and C2-CP, we determined that SCNPC tumors had significant alterations in 4 MET-associated gene sets in the UW cohort (p<0.05; **S2A Fig**) and in 5 MET-associated gene sets in the SU2C cohort (p<0.05; **S2B Fig**) compared to adenocarcinomas. This analysis suggests that *MET* expression and downstream signaling may impact tumor progression in SCNPC.

Next, we leveraged the RNA-Seq data to examine the expression of other known cabozantinib targets including *RET*, *VEGFR2/KDR*, *AXL*, *KIT* and *TEK* [11,21]. Importantly, *RET* expression was significantly upregulated in SCNPC tumors in both the UW (p<0.0001) and SU2C (p<0.0001) datasets compared to AR-high metastases, whereas *RET* expression was low in AR-/NE- (i.e. double-negative prostate cancer; [19]) and AR-low metastases, indicating that *RET* expression is not linked to AR-loss (**Figs 1A, 1B, S3A and S3B**). In parallel, *RET* expression was weakly negatively correlated with AR activity score in the UW dataset (Pearson’s correlation r = -0.27; p = 0.0078), but was not correlated with AR activity score in the SU2C dataset (Pearson’s correlation r = 0.051; p = 0.4; **S3C and S3D Fig**). GSEA revealed that RET signaling was significantly enriched in SCNPC tumors compared to adenocarcinomas in both UW and SU2C cohorts (p<0.01; **S3E Fig**). While the expression of *VEGFR2*/*KDR*, *AXL* and *TEK* did not have clear associations with AR activity or the SCNPC phenotype, *KIT* expression was also upregulated in SCNPC in both the UW and SU2C datasets (**Figs 1A, 1B and S4**). Taken together, these data supported *MET* and *RET* expression as potential biomarkers for stratifying SCNPC tumors for cabozantinib therapy.

### Cabozantinib activity in experimental models of SCNPC

**Fig 2:**
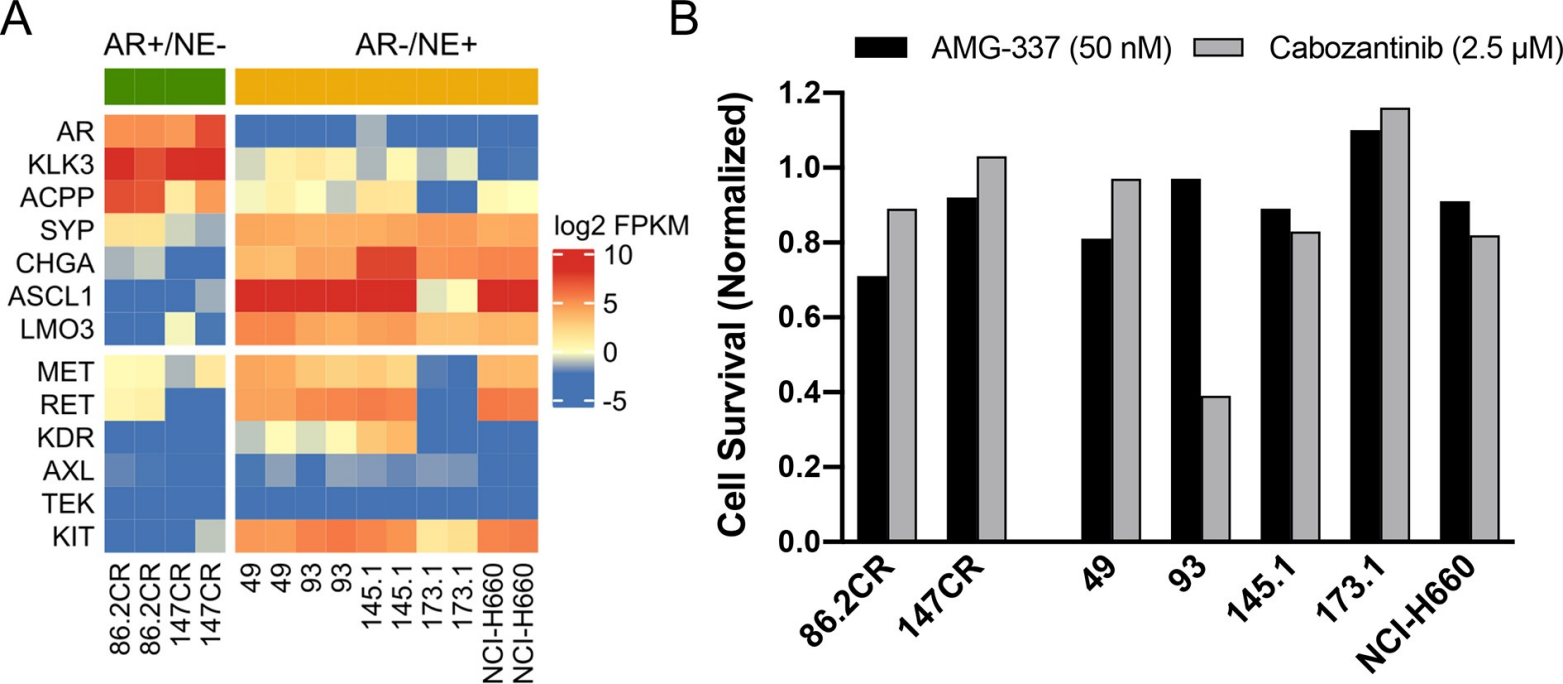
The identification of *MET* and *RET* expressing SCNPC PDX models and responses to AMG-337 and cabozantinib *in vitro*. (A) RNA-Seq heatmap of LuCaP PDX models and NCI-H660 cells showing AR-associated genes (*AR*, *KLK3* and *ACPP*), NE-associated genes (*SYP*, *CHGA*, *ASCL1* and *LMO3*), targets of cabozantinib (*MET*, *RET*, *KDR*, *AXL*, *TEK*, *KIT*). Results are expressed as log_2_ FPKM and are colored according to scale. (B) Cell viability assay of dissociated LuCaP PDX tumors and NCI-H660 cells. Cells were treated with vehicle control, AMG-337 (50 nM) or cabozantinib (2.5 μM) for 72h. Results are expressed as percent viable cells and are normalized to vehicle treated controls.

To further interrogate the translational potential of *MET* and *RET* expression and cabozantinib treatment in SCNPC, we evaluated two adenocarcinoma LuCaP CRPC PDX models (LuCaP 86.2CR and LuCaP 147CR), 4 SCNPC PDX models (LuCaP 49, 93, 145.1 and 173.1) and the SCNPC NCI-H660 cell line. Similar to the UW and SU2C mCRPC cohorts, transcriptome analysis revealed that AR-active adenocarcinoma PDX models had negligible *MET* and *RET* transcript expression, whereas 3 of 4 SCNPC PDX models and the NCI-H660 cell line had robust *MET* and *RET* expression (**Fig 2A**). To determine if MET and RET expression promotes cell survival, we conducted *in vitro* drug screens using the MET inhibitor AMG-337 [22,23] and cabozantinib. Of note, AMG-337 had limited effects on cell viability and only LuCaP 93 responded to cabozantinib with a > 50% decrease in viable cells relative to control after three days of treatment (**Fig 2B**). Based on the differential expression of *MET* and *RET* and the response to cabozantinib treatment *in vitro*, we selected LuCaP 93 (MET+/RET+) and LuCaP 173.1 (MET-/RET-) PDX models for *in vivo* studies.

**Fig 3:**
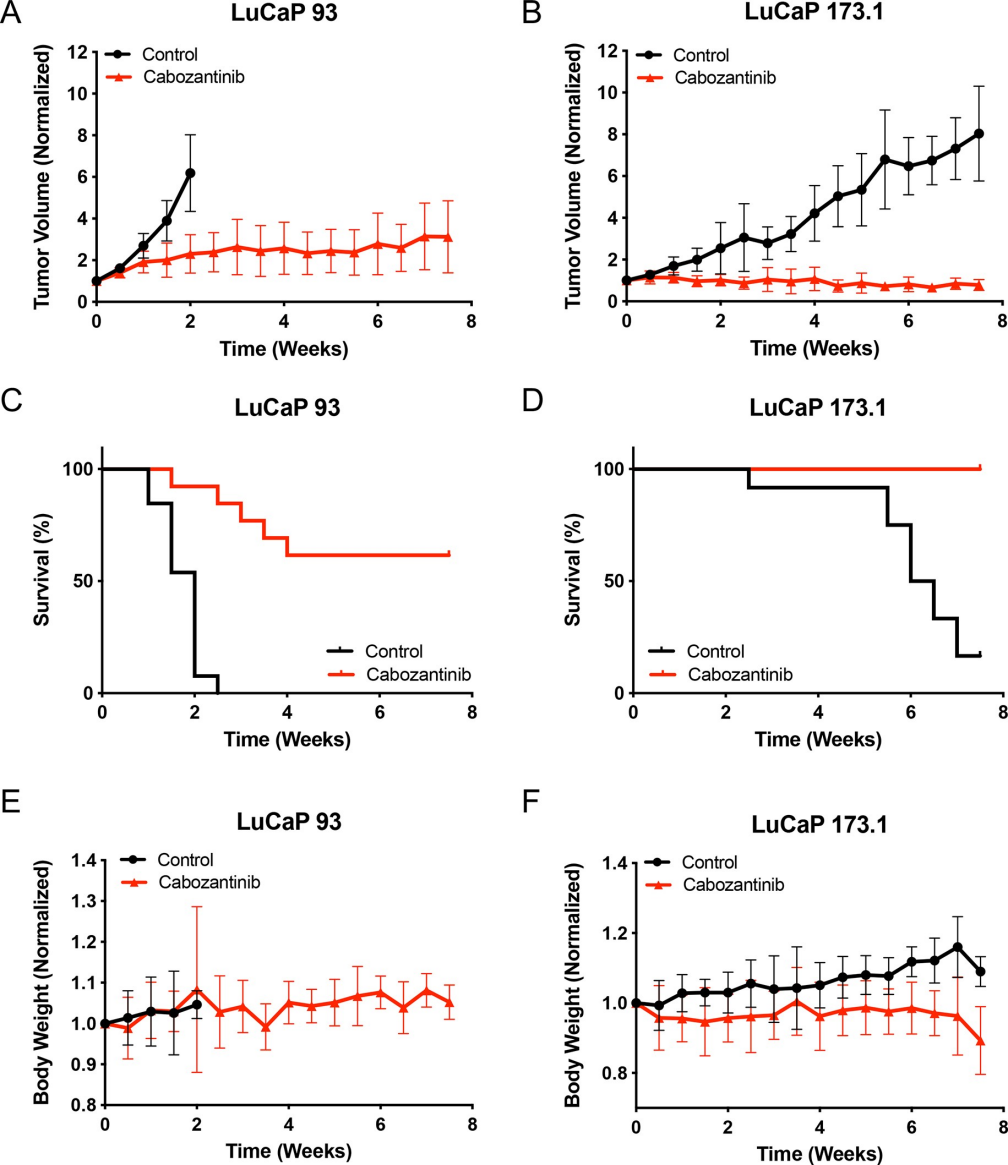
Cabozantinib significantly decreases tumor volume and prolongs survival in SCNPC PDX models. SCNPC PDX models were treated with 30 mg/kg of cabozantinib (LuCaP 93, n = 14; LuCaP 173.1 n = 14) or vehicle control (LuCaP 93, n = 14; LuCaP 173.1, n = 14) for up to 7.5 weeks. (A) LuCaP 93 and (B) LuCaP 173.1 normalized tumor volume measurements. LuCaP 93, p-value = 0.0175; LuCaP 173.1, p-value <0.0001. (C) LuCaP 93 and (D) LuCaP 173.1 overall survival curves using Kaplan-Meier analysis and the log-rank (Mantel-Cox) test. LuCaP 93, p-value < 0.0001; LuCaP 173.1, p-value < 0.0001. (E) LuCaP 93 and (F) LuCaP 173.1 normalized body weight measurements. LuCaP 93, p-value = 0.1677; LuCaP 173.1, p-value <0.0002. Black = control vs. red = cabozantinib.

In animals bearing LuCaP 93 tumors, cabozantinib treatment significantly decreased tumor volume (TV) in MET+/RET+ LuCaP 93 animals compared to vehicle controls (p = 0.0175; **Fig 3A**). Due to the rapid growth of LuCaP 93 PDX tumors, all control animals were sacrificed at two weeks. Interestingly, and in contrast to the *in vitro* results, cabozantinib treatment significantly decreased TV in MET-/RET- LuCaP 173.1 compared to vehicle controls (p<0.0001; **Fig 3B**). In addition, cabozantinib-treated animals had significantly longer survival times (LuCaP 93, p<0.0001; LuCaP 173.1, p<0.0001) compared to control animals (**Fig 3C and 3D**). Finally, cabozantinib treatment had no significant effect on body weight of LuCaP 93 (p = 0.1677), but decreased body weight of LuCaP 173.1 (p = 0.0002) tumor bearing animals compared to control animals (**Fig 3E and 3F**). Together, these data suggest that cabozantinib inhibits SCNPC tumor growth *in vivo* irrespective of tumor *MET* and *RET* status.

### Cabozantinib inhibits microvessel formation and increases tumor hypoxia in SCNPC PDX tumors

**Fig 4:**
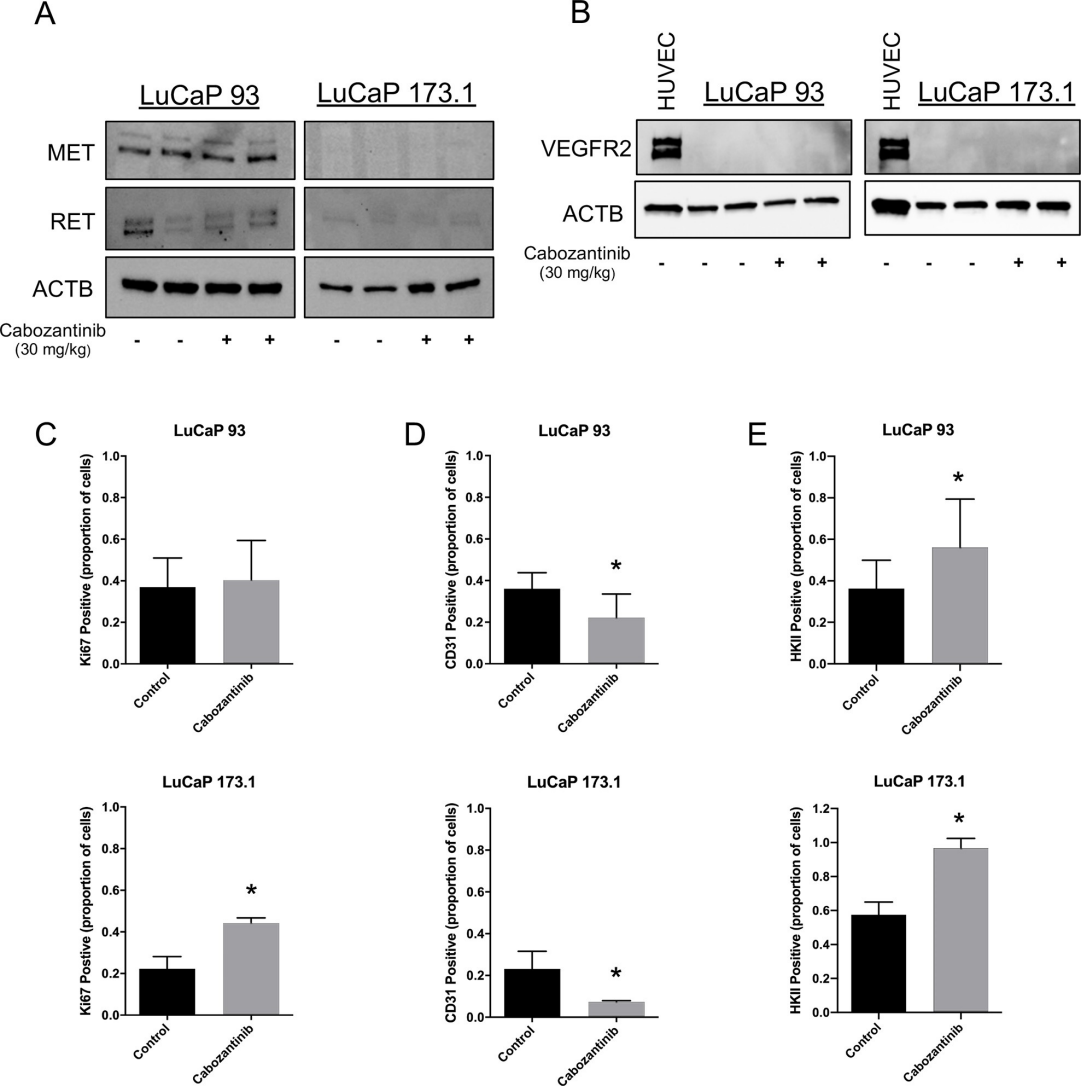
Cabozantinib treatment decreased microvessel density, but not proliferation and increased hypoxic stress in LuCaP 93 and LuCaP 173.1 patient-derived xenografts. (A) Immunoblots of tumor lysates from LuCaP 93 and LuCaP 173.1 *in vivo* study tumors. Blots were probed with primary antibodies to MET, RET and ACTB. (B) Immunoblots of HUVEC cells and tumor lysates from LuCaP 93 and LuCaP 173.*1 in vivo* study tumors. HUVEC cell lysates were used as a positive control and blots were probed with primary antibodies to VEGFR2 and ACTB. (C) Graphs of Ki67 positive cells in control vehicle and cabozantinib-treated LuCaP 93 (p = 0.63, top graph) and 173.1 (p<0.001, bottom graph)* tumors. (D) Graphs of CD31 positive cells in control vehicle and cabozantinib-treated LuCaP 93 (p = 0.001; top graph) and LuCaP 173.1 (p = 0.01; bottom graph) tumors. (E) Graphs of Hexokinase II positive cells in control vehicle and cabozantinib-treated LuCaP 93 (p = 0.0184; top graph) and LuCaP 173.1 (p<0.001; bottom graph) tumors. Results are plotted as mean ± SD. *The cabozantinib-treated group in the LuCaP 173.1 study had only 3 tumors that could be assessed as the remaining tumors were too necrotic or too small to assess.

To validate the RNA-Seq of *MET* and *RET* transcript expression in LuCaP 93 and LuCaP 173.1, we conducted immunoblot analysis on *in vivo* study tumors to determine MET and RET protein status. In agreement with the RNA-Seq results, MET and RET proteins were expressed in LuCaP 93 in both control and cabozantinib-treated tumors (**Fig 4A**). Moreover, MET protein expression was absent in LuCaP 173.1 in both control and treated tumors (**Fig 4A**). Importantly, immunoblot detected very low RET expression in LuCaP 173.1 tumors but we used a RET primary antibody that cross-reacts with mouse RET and the observed RET protein expression is likely derived from murine immune cells associated with the tumor microenvironment [24]. Mirroring the RNA-Seq profiles, VEGFR2 protein expression was absent in all LuCaP 93 and LuCaP 173.1 study tumors, whereas VEGFR2 protein was strongly expressed in positive control HUVEC cells (**Fig 4B**). Immunohistochemical (IHC) analysis of treatment resistant-tumors obtained at the end of the study revealed that proliferation (Ki67 staining) was not significantly decreased in cabozantinib-treated LuCaP 93 and LuCaP 173.1 tumors relative to controls (**Fig 4B**). In fact, an increase in Ki67 staining was observed in treatment-resistant tumors of LuCaP 173.1 only. However, cabozantinib significantly decreased microvessel density (CD31 staining) in both LuCaP 93 (p = 0.001) and LuCaP 173.1 (p = 0.01) PDX tumors relative to tumors from control animals (**Figs 4C and S5A**). In addition, Hexokinase II, a hypoxia-inducible biomarker of glycolysis [25,26], was significantly increased in cabozantinib-treated LuCaP 93 (p = 0.0184) and LuCaP 173.1 (p < 0.001) tumors compared to control vehicle-treated tumors (**Figs 4D and S5B**). These data suggest that cabozantinib, a VEGFR2 inhibitor, could inhibit SCNPC tumor growth through disruption of the tumor vasculature *in vivo*.

**Fig 5:**
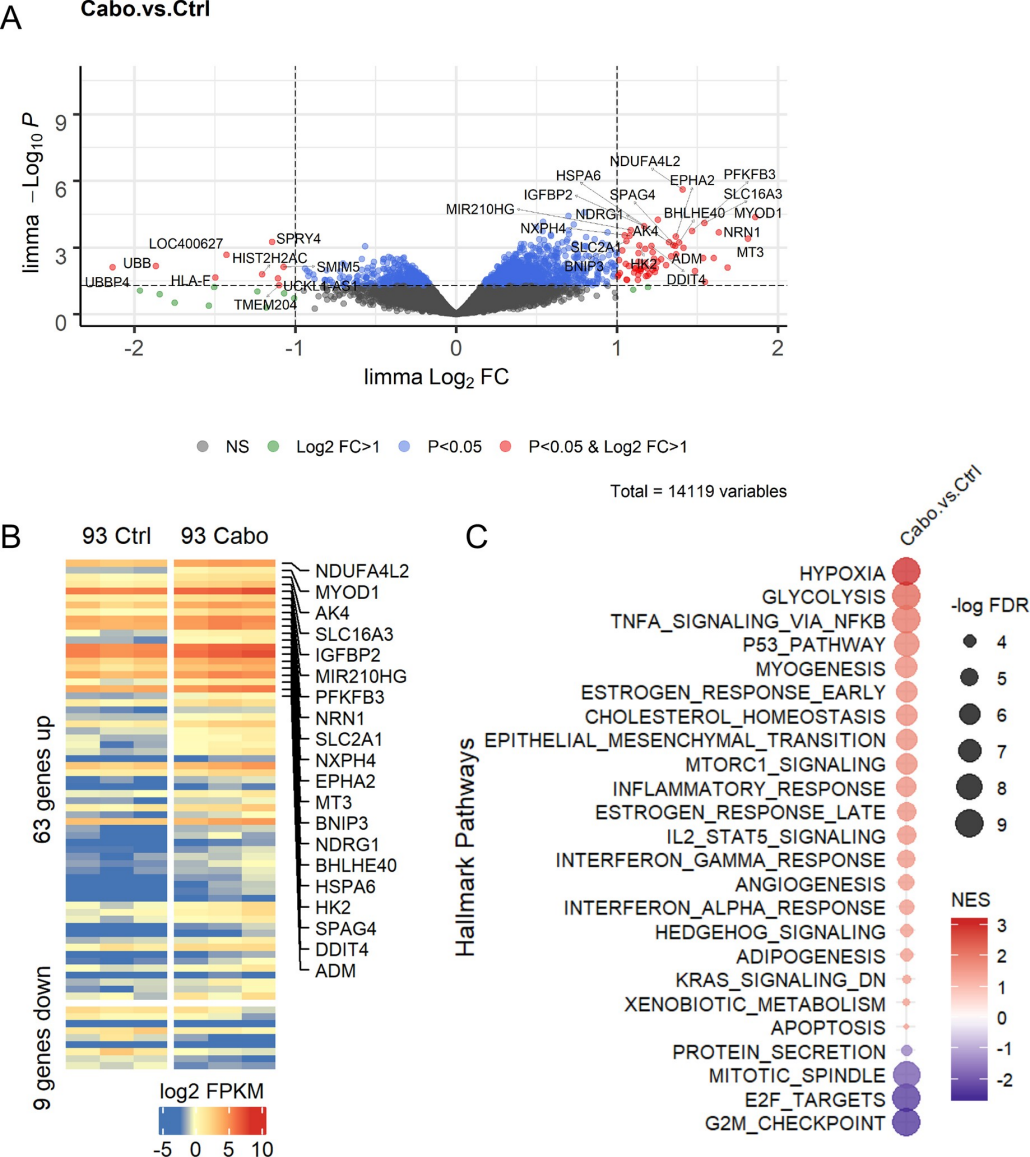
RNA-Seq and pathway analysis of LuCaP 93 tumors from cabozantinib-treated animals compared to tumors from vehicle treated animals. RNA was isolated for RNA-Seq from vehicle control (n = 3) and cabozantinib-treated (n = 3) LuCaP 93 tumor bearing animals. (A) Volcano plot representing up- and downregulated genes in response to cabozantinib treatment. Statistically significant changes in gene expression were based on limma and only included genes with p<0.05 and log_2_ Fold Change (FC)>1. Data points are colored according legend; NS = not significant. (B) Unsupervised clustering heatmap of significantly altered genes in response to cabozantinib treatment in LuCaP 93 PDX tumors. The top 20 upregulated genes are labeled. Data represents log_2_ FPKM values and are colored according to scale. (C) GSEA of significantly altered genes from (B) using the hallmark gene sets in MSigDB. Ctrl: vehicle control; Cabo: cabozantinib.

To determine the molecular effects of cabozantinib treatment on LuCaP PDX models, we used RNA-Seq and gene set enrichment analysis to identify genes and biological pathways altered in treated LuCaP 93 tumors. There was insufficient material with a viable RNA integrity number available from LuCaP 173.1 study tumors for RNA-Seq. Nevertheless, transcriptome analysis of LuCaP 93 tumors collected after 5 days of cabozantinib treatment identified significantly altered genes relative to control tumors (**Fig 5A and 5B**). In agreement with the microvessel density and HK2 data, 9 of the top 10 upregulated genes in cabozantinib-treated tumors are known to be hypoxia-inducible or are directly regulated by hypoxia-inducible factors (**Fig 5B**; Refs [27–34]). Indeed, gene ontology and pathway analysis of significantly up and downregulated genes identified hypoxia and glycolysis as two major pathways that were active in cabozantinib-treated tumor cells, indicative of VEGFR2 inhibition (**Fig 5C**). While Ki67 staining suggested that there were no differences in 5-day cabozantinib-treated tumors compared to control tumors (percent positive cells = 0.50±0.03 vs 0.50±0.05, p = 0.93, n = 3), proliferation associated pathways such as E2F targets and G2M checkpoint pathways, were decreased in the cabozantinib-treated tumor cells (**Fig 5C**). Of note, GSEA using 17 MET-associated gene sets determined that none of the gene sets were significantly altered in cabozantinib treatment vs control tumors (**S6A Fig**). However, one of three RET-associated pathways was altered in cabozantinib-treated tumors (**S6B Fig**). Taken together, these results suggest that cabozantinib treatment drastically increased tumor-associated hypoxic stress, and tumor growth inhibition likely occurred independent of MET pathway inhibition in the LuCaP 93 PDX model.

## Discussion

An increase in the AR-null SCNPC phenotype of mCRPC in the context of resistance to first- and second-line AR signaling inhibitors has been reported by our group and others [1,3,19]. A recent NCI workshop on Lineage Plasticity in AR-independent prostate cancer highlighted an urgent need to identify novel therapeutic targets to treat CRPC that bypasses AR-directed therapies [35]. Both MET and RET have been proposed as targets for treatment in patients with CRPC that display a decrease in AR expression or have converted to a SCNPC phenotype respectively [4–6]. In this report, we confirmed that elevated levels of *MET* and *RET* transcripts were associated with the SCNPC phenotype in patient metastases from our rapid autopsy program [2] and in the SU2C/PCF cohort [16], and similar characteristics were observed in LuCaP SCNPC PDX models. Thus, we considered a rational approach for targeting elevated levels of MET and RET in SCNPC through the use of cabozantinib [21]. Cabozantinib clinical trials in men with heavily pre-treated mCRPC did not attain the primary end points of better overall survival or improved pain response [8,9]. However, the patients in these studies were not selected for SCNPC phenotypes nor for *MET* expression which would serve as a logical biomarker to predict benefit from cabozantinib therapy [10].

Previous findings by our group demonstrated that cabozantinib inhibited the growth of AR-expressing CRPC and altered bone remodeling elicited by prostate cancer cells *in vivo* [36]. However, leveraging the insights gleaned from cabozantinib clinical trials, we tried to model the impact of cabozantinib on the progression of SCNPC. We identified LuCaP 93 and LuCaP 173.1 as PDX models that would differentiate the response of a MET+/RET+ tumor from a MET-/RET- tumor to cabozantinib treatment. The *in vitro* cabozantinib screen was aligned with MET and RET expression and only showed efficacy in MET and RET positive LuCaP 93 cells, demonstrating that cabozantinib can elicit direct effects on SCNPC tumor cells driven by MET or RET activity. However, *in vivo* cabozantinib treatment significantly inhibited tumor growth and prolonged the survival of both LuCaP 93 and LuCaP 173.1. IHC assessment of the tumors demonstrated that cabozantinib significantly decreased microvessel density and increased hypoxic stress in both PDX models, whereas proliferation was not decreased. In parallel, RNA-Seq determined that there was an increase in hypoxia-associated genes and hypoxia and glycolysis pathways by GSEA in LuCaP 93 cabozantinib-treated tumors.

Cabozantinib displays potent activity towards blocking VEGFR2 activity [21]. Notably, cabozantinib therapy is approved for renal cell carcinoma and the METEOR trial demonstrated improvements in OS in patients who have progressed on first-line VEGFR inhibitors [37]. VEGFR2 activity promotes a response to VEGF and regulates endothelial cell migration, proliferation and tumor vascularization [38]. Despite increased MET and RET expression in SCNPC, our results cumulatively suggest that cabozantinib acted through mouse endothelial VEGFR2 and inhibited angiogenesis leading to hypoxia and tumor cell death regardless of MET/RET status in both LuCaP SCNPC PDX models. In support of this hypothesis, cabozantinib inhibits VEGFR2 and endothelial cell tube formation *in vitro* in both mouse and human endothelial cell models [39,40]. While our study is the first to examine the efficacy of cabozantinib in SCNPC, other preclinical prostate cancer studies in AR-active CRPC agree that VEGFR2 inhibition and disruption of the tumor vasculature could be principal components of cabozantinib-mediated tumor growth inhibition [36,41,42]. This does not negate the possible direct effect of cabozantinib on MET and RET activity in the tumor cells of the MET+/RET+ LuCaP 93 xenografts. However, AMG-337 treatments had limited effects *in vitro* and GSEA on cabozantinib-treated LuCaP 93 tumors compared to control tumors revealed no significantly altered MET-associated pathways and significant changes in only one of three RET-associated pathways. Congruently, targeting MET in CRPC clinical trials has shown no anticancer activity [43], suggesting that MET activity is not a primary driver of tumor progression in prostate cancer patients.

Tumor vascularization can be divided into either a tumor vessel phenotype with vessels distributed amongst the tumor cells or a stromal vessel phenotype with tumor cells enveloped by intricate matrices of vessels and stroma [44]. Furthermore, the tumor vessel phenotype predicts response to VEGF-directed therapies [44]. Whether response to cabozantinib therapy can be discriminated by vascular phenotypes and if SCNPC is a model of the responsive tumor vessel phenotype are open questions that warrant further investigation. Of note, CRPC metastases display considerable heterogeneity in angiogenic capacities but bone and lymph node tumors contain significantly higher vessel density and distribution compared to liver metastases [45]. Indeed, secondary endpoints of the COMET-1 trial determined that cabozantinib treatment significantly decreased circulating tumor cells and improved bone-specific parameters [8]. These data suggest that the true clinical activity of cabozantinib is likely through disruption of tumor microvessel structure and provide further support for the hypothesis that the primary response to cabozantinib in our SCNPC studies was mediated through tumor endothelial cell VEGFR2 inhibition.

Recent reports suggest that induction of FGFR1 expression is a mechanism of cabozantinib resistance in CRPC [42,46]. It is well documented that FGF signaling plays a critical role in tumor vascularization and that FGFR amplification can bypass VEGFR-directed therapies in multiple cancer types [47]. Moreover, expression of FGFR1 is associated with transition to CRPC, and the FGF pathway can drive tumor progression in tumors refractory to AR-directed therapies [19,48]. Thus, future preclinical studies investigating cabozantinib as a combination therapy with FGF pathway inhibitors could translate to potential therapies for patients with AR-independent tumors.

In conclusion, our data indicate that preventing tumor vascularization is the predominant mechanism of cabozantinib-mediated tumor inhibition in two PDX models of SCNPC. Addressing the activity of cabozantinib on the existing stromal architecture in established tumors remains to be determined. However, cabozantinib may represent a potential therapy for patients with metastatic disease in tumor phenotypes that have a significant dependence on the tumor vasculature for survival and proliferation.

## Supporting information

S1 Fig MET expression in the five molecular phenotypes of mCRPC. Analysis of RNA-Seq from (A) UW rapid autopsy (n = 98) and (B) SU2C (n = 270) mCRPC cohorts for significantly altered *MET* expression. AR+/NE- = AR-high PC; AR+/NE+ = Amphicrine PC; AR^low^/NE- = AR-low PC; AR-/NE- = Double-negative PC; AR-/NE+ = Small cell or neuroendocrine PC. P-values = ****p < 0.0001; ns = not significant; 1-way ANOVA with Tukey’s multiple comparisons test.
(TIF)

S2 Fig GSEA reveals altered MET activity in SCNPC tumors compared to adenocarcinomas. Analysis of (A) UW rapid autopsy cohort and (B) SU2C cohort for significantly altered MET-associated gene sets from C2 in MSigDB. Each datapoint in the boxplots represent a single tumor. Adenocarcinoma (AR+/NE-, green); SCNPC (AR-/NE+, yellow). P-values = *: p < 0.05; **: p < 0.01; ***: p < 0.001; ****: p < 0.0001.
(TIF)

S3 Fig RET expression is upregulated in SCNPC and does not associate with AR activity in mCRPC. Analysis of RNA-Seq from (A) UW rapid autopsy (n = 98) and (B) SU2C (n = 270) mCRPC cohorts for significantly altered *RET* expression. Analysis of RNA-Seq from (C) UW rapid autopsy (n = 98) and (D) SU2C (n = 270) mCRPC cohorts for associations with RET expression and AR activity. r-values were determined through a Pearson’s correlation analysis. (E) Significantly altered RET-associated gene sets from C2 in MSigDB. Each datapoint in the boxplots represent a single tumor. Adenocarcinoma (AR+/NE-, green); SCNPC (AR-/NE+, yellow). P-values = *: p < 0.05; **: p < 0.01; ***: p < 0.001; ****: p < 0.0001.
(TIF)

S4 Fig Correlation of VEGFR2 expression with AR signaling in SU2C and UWRA mCRPC datasets. Analysis of RNA-Seq from (A) UW rapid autopsy (n = 98) and (B) SU2C (n = 270) mCRPC cohorts for associations with KDR/VEGFR2 expression and AR activity. r-values were determined through a Pearson’s correlation analysis.
(TIF)

S5 Fig Cabozantinib treatment decreases microvessel density and increases hypoxic stress in SCNPC PDX models. Immunohistochemistry of (A) CD31 and (B) hexokinase II using representative LuCaP 93 and 173.1 tumor specimens. Arrows indicate CD31 positive vessels in A or Hexokinase II positive cells in B. Scale bars: 20 μm.
(TIF)

S6 Fig Cabozantinib treatment does not significantly alter MET-associated pathways in LuCaP 93. **(**A) Boxplots of GSVA enrichment scores for control and cabozantinib-treated tumors for 17 MET-associated gene sets from C2 in MSigDB. (B) Boxplots of GSVA enrichment scores for control and cabozantinib-treated tumors displaying RET-associated gene sets from C2 in MSigDB. Each datapoint in the boxplots represent a single tumor. Ctrl: vehicle control, grey dots; Cabo: cabozantinib, black dots. P-values = *: p < 0.05; ns = not significant.
(TIF)

S1 Raw images (PDF)
