# Supplementary figures and images for: Cabozantinib can block growth of neuroendocrine prostate cancer patient-derived xenografts by disrupting tumor vasculature
Source: PLoS One. 2021 Jan 20;16(1):e0245602. doi: 10.1371/journal.pone.0245602 (PMC7817027; doi:10.1371/journal.pone.0245602)

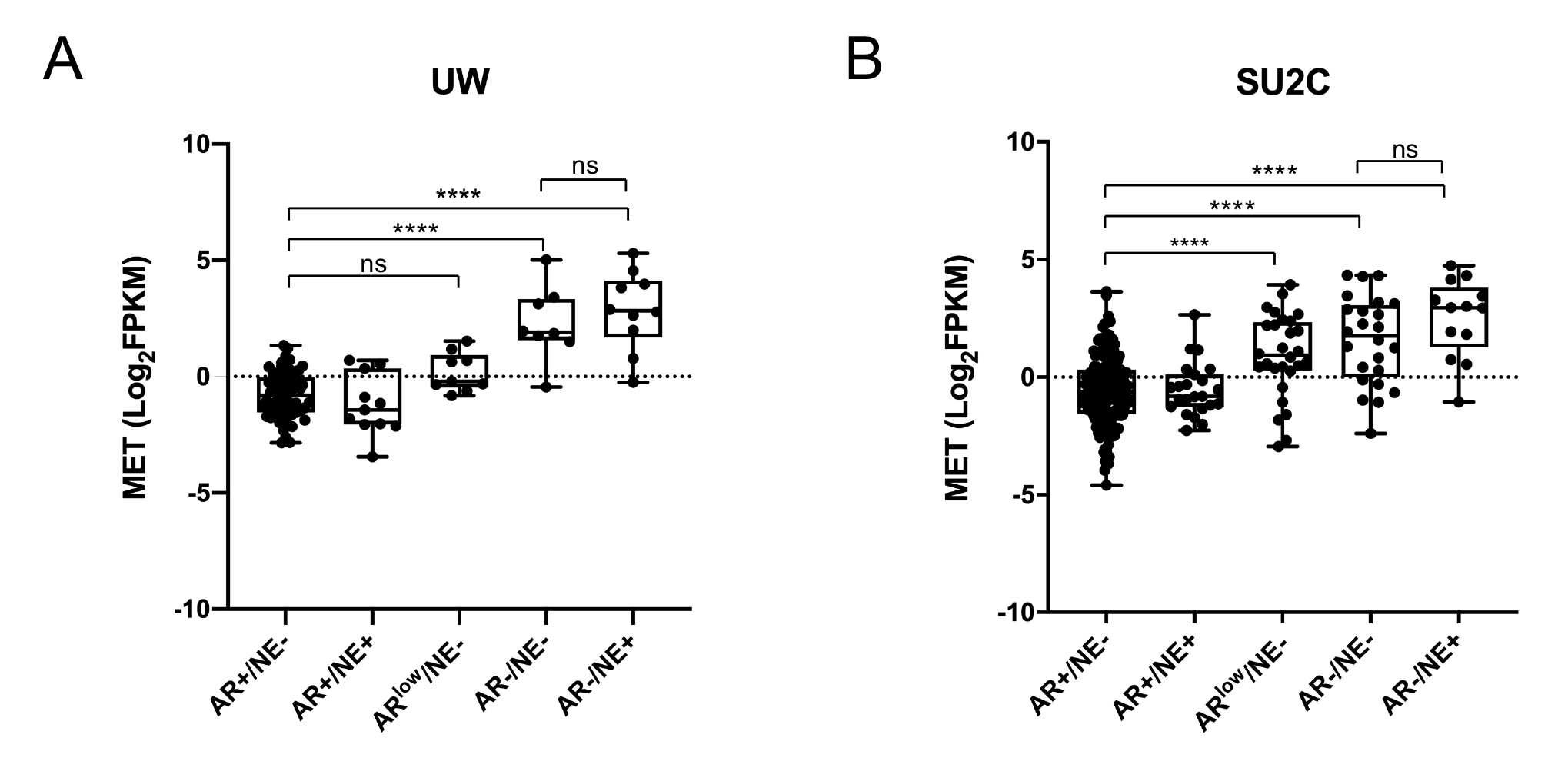

Supplement: S1 Fig — Analysis of RNA-Seq from (A) UW rapid autopsy (n = 98) and (B) SU2C (n = 270) mCRPC cohorts for significantly altered MET expression. AR+/NE- = AR-high PC; AR+/NE+ = Amphicrine PC; ARlow/NE- = AR-low PC; AR-/NE- = Double-negative PC; AR-/NE+ = Small cell or neuroendocrine PC. P-values = ****p < 0.0001; ns = not significant; 1-way ANOVA with Tukey’s multiple comparisons test. (TIF) [file pone.0245602.s001.tif]

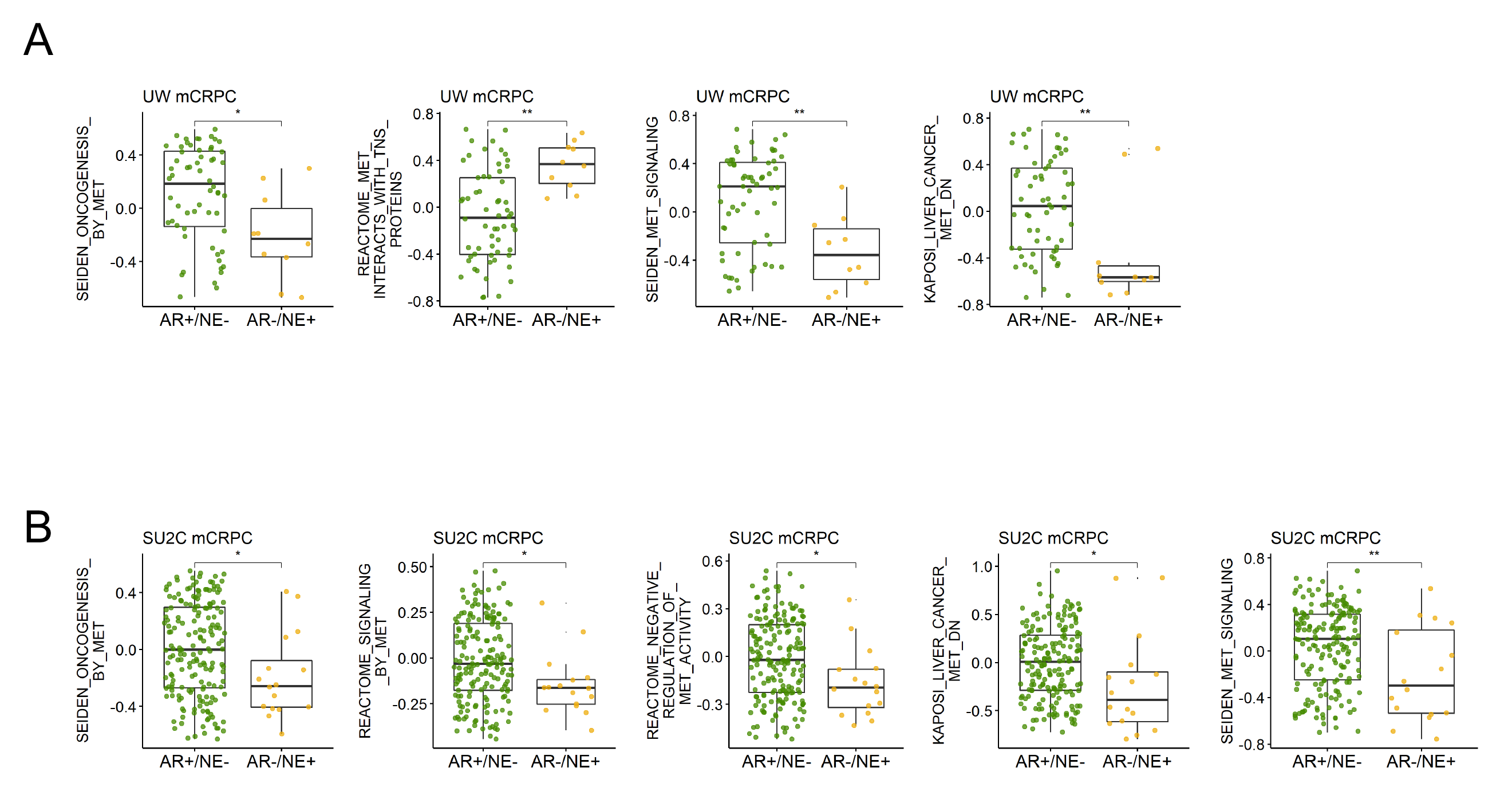

Supplement: S2 Fig — Analysis of (A) UW rapid autopsy cohort and (B) SU2C cohort for significantly altered MET-associated gene sets from C2 in MSigDB. Each datapoint in the boxplots represent a single tumor. Adenocarcinoma (AR+/NE-, green); SCNPC (AR-/NE+, yellow). P-values = *: p < 0.05; **: p < 0.01; ***: p < 0.001; ****: p < 0.0001. (TIF) [file pone.0245602.s002.tif]

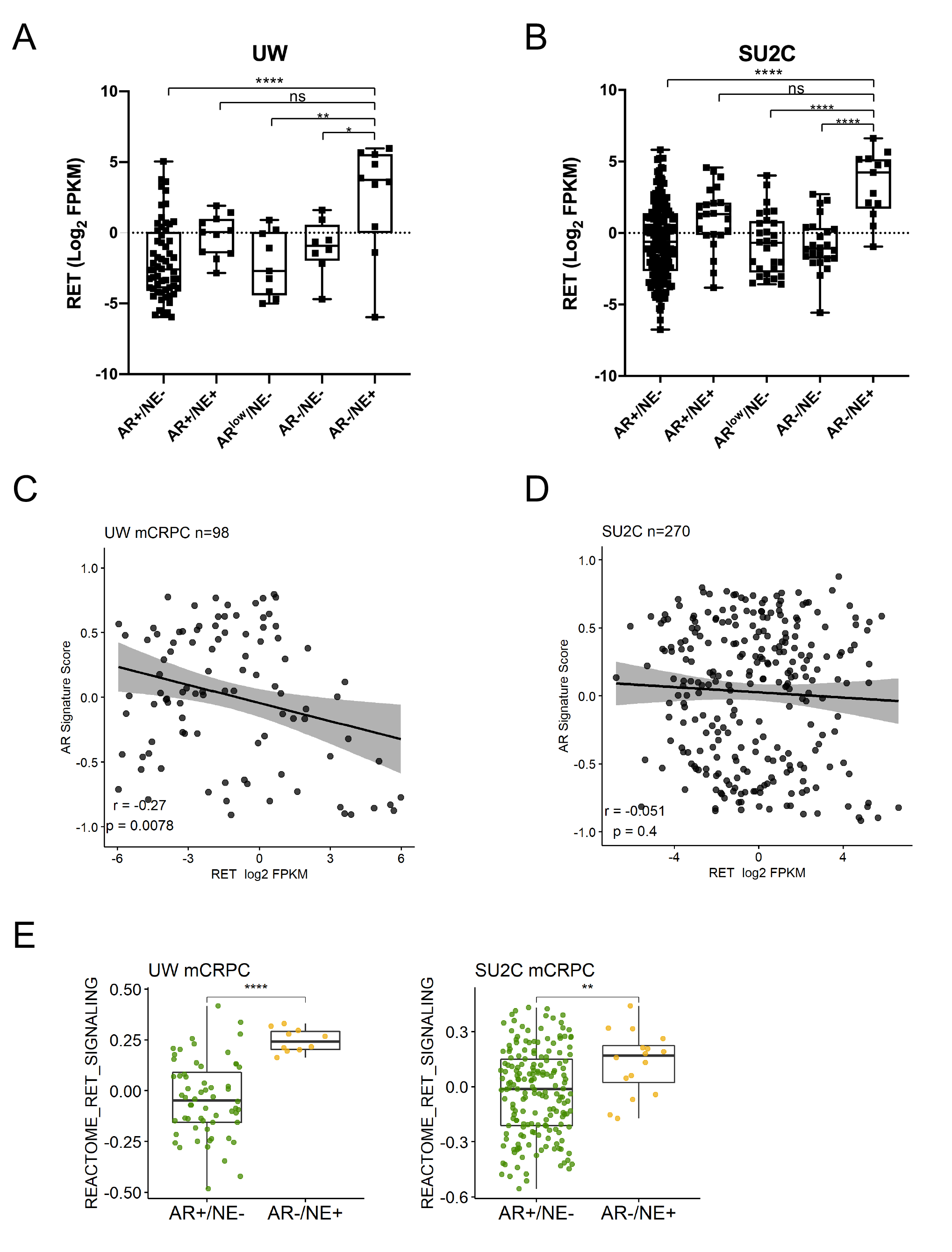

Supplement: S3 Fig — Analysis of RNA-Seq from (A) UW rapid autopsy (n = 98) and (B) SU2C (n = 270) mCRPC cohorts for significantly altered RET expression. Analysis of RNA-Seq from (C) UW rapid autopsy (n = 98) and (D) SU2C (n = 270) mCRPC cohorts for associations with RET expression and AR activity. r-values were determined through a Pearson’s correlation analysis. (E) Significantly altered RET-associated gene sets from C2 in MSigDB. Each datapoint in the boxplots represent a single tumor. Adenocarcinoma (AR+/NE-, green); SCNPC (AR-/NE+, yellow). P-values = *: p < 0.05; **: p < 0.01; ***: p < 0.001; ****: p < 0.0001. (TIF) [file pone.0245602.s003.tif]

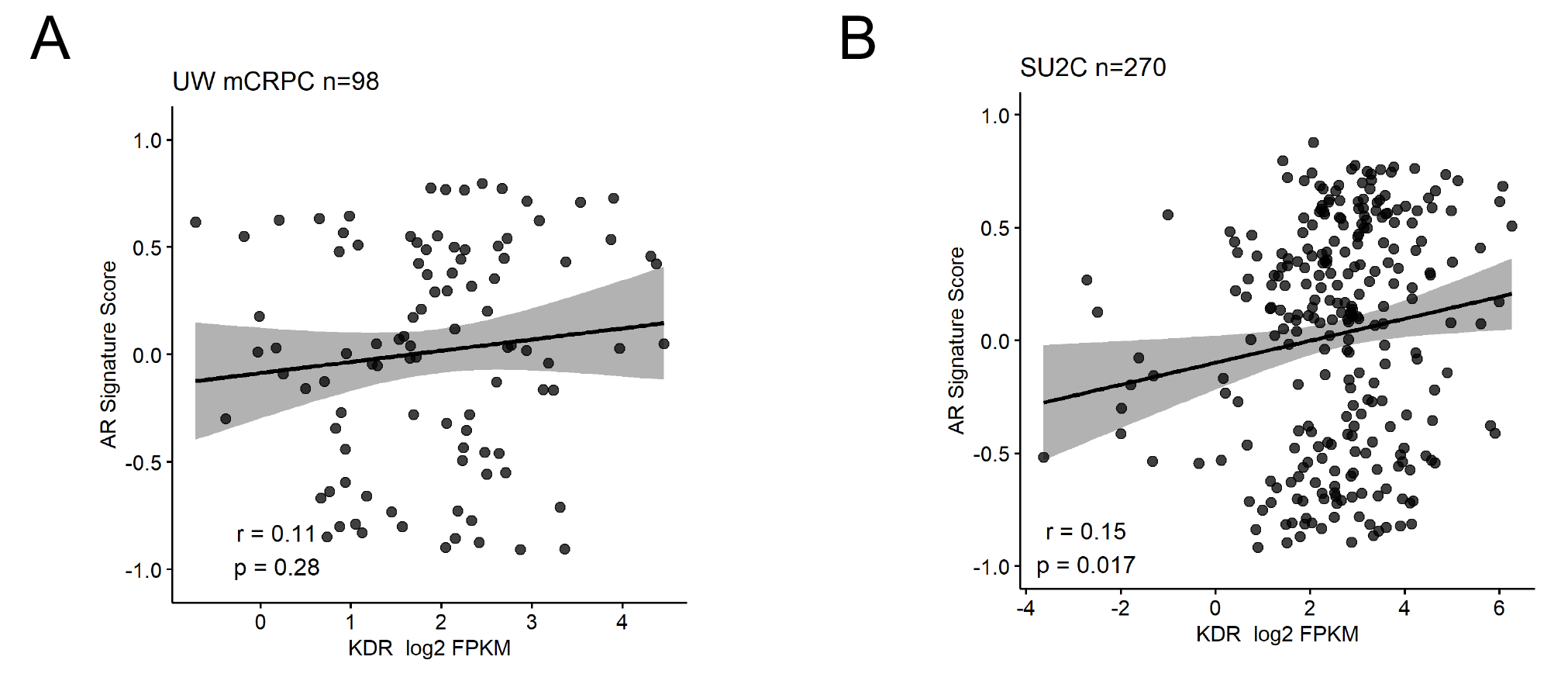

Supplement: S4 Fig — Analysis of RNA-Seq from (A) UW rapid autopsy (n = 98) and (B) SU2C (n = 270) mCRPC cohorts for associations with KDR/VEGFR2 expression and AR activity. r-values were determined through a Pearson’s correlation analysis. (TIF) [file pone.0245602.s004.tif]

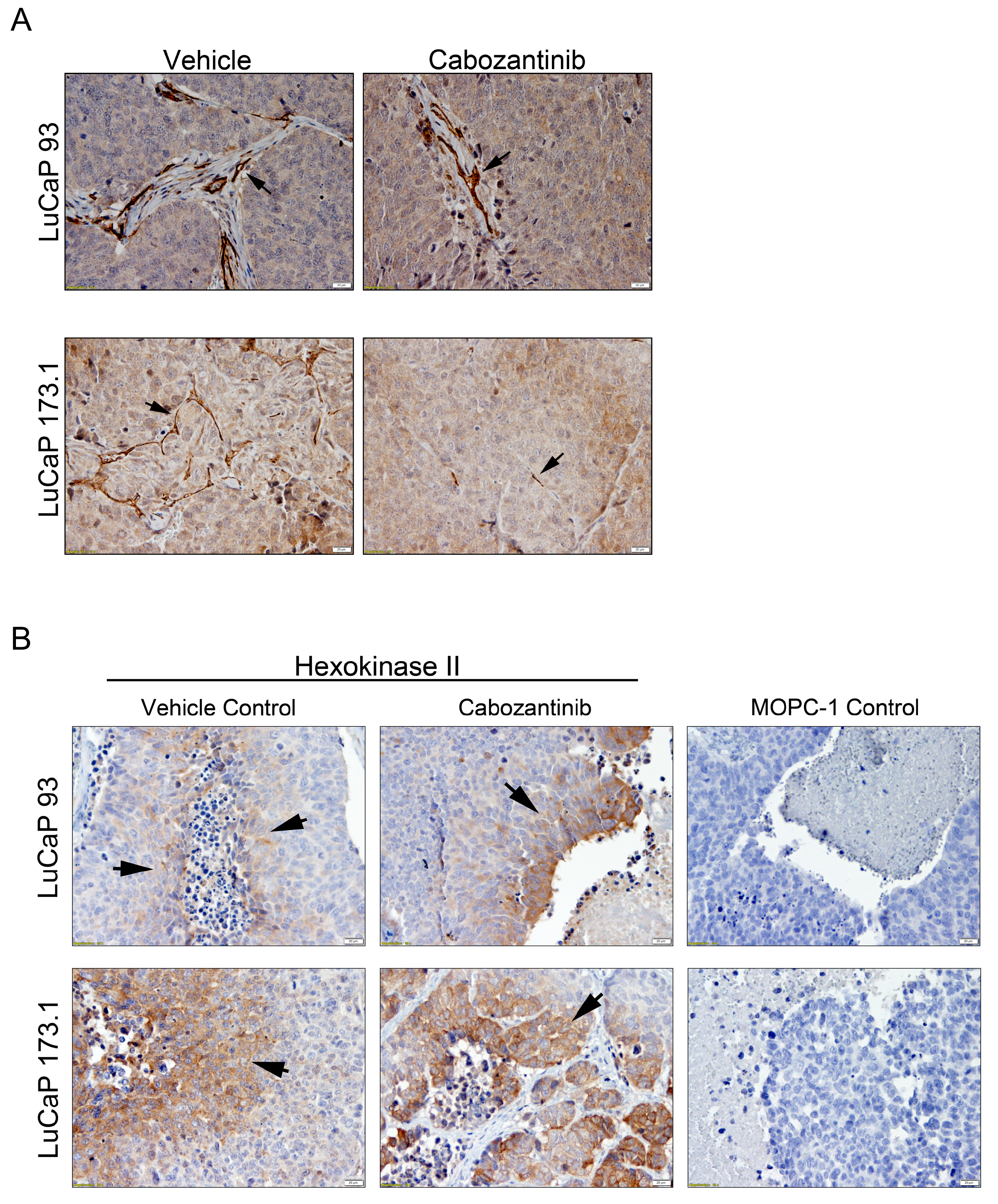

Supplement: S5 Fig — Immunohistochemistry of (A) CD31 and (B) hexokinase II using representative LuCaP 93 and 173.1 tumor specimens. Arrows indicate CD31 positive vessels in A or Hexokinase II positive cells in B. Scale bars: 20 μm. (TIF) [file pone.0245602.s005.tif]

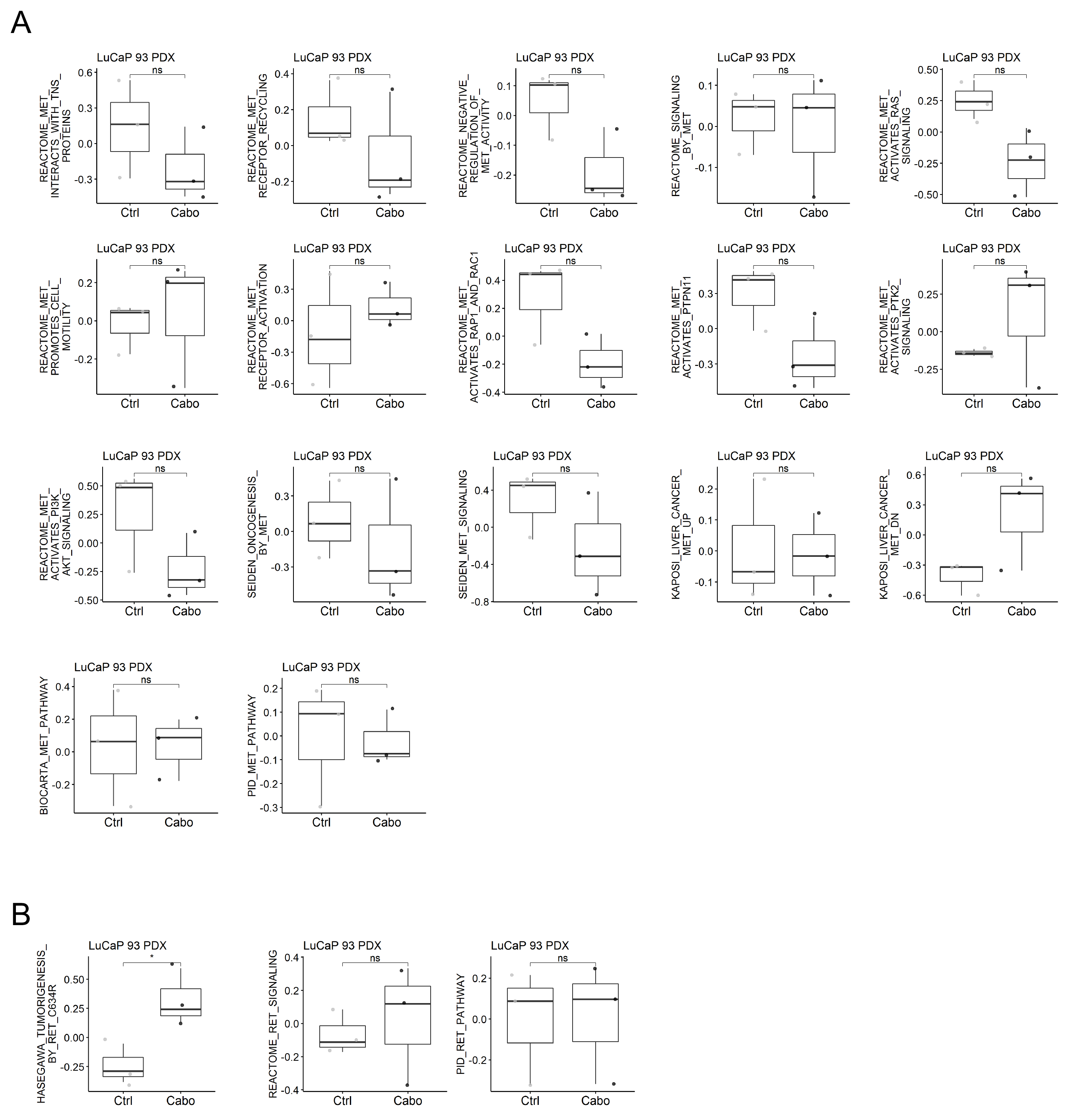

Supplement: S6 Fig — (A) Boxplots of GSVA enrichment scores for control and cabozantinib-treated tumors for 17 MET-associated gene sets from C2 in MSigDB. (B) Boxplots of GSVA enrichment scores for control and cabozantinib-treated tumors displaying RET-associated gene sets from C2 in MSigDB. Each datapoint in the boxplots represent a single tumor. Ctrl: vehicle control, grey dots; Cabo: cabozantinib, black dots. P-values = *: p < 0.05; ns = not significant. (TIF) [file pone.0245602.s006.tif]

Figure 4A – Western Blots

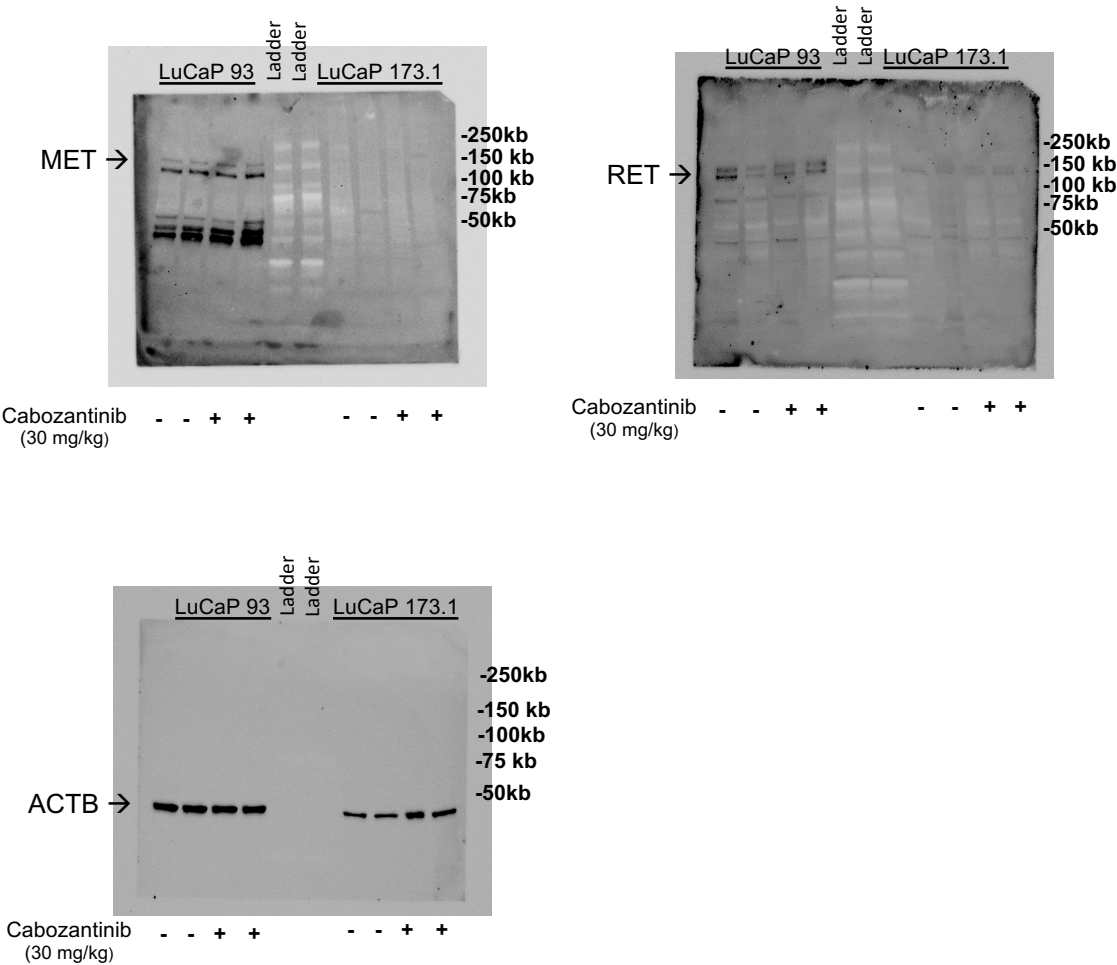

Figure 4B – Western Blots

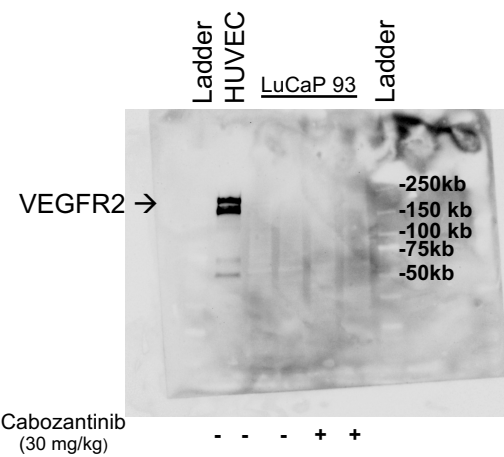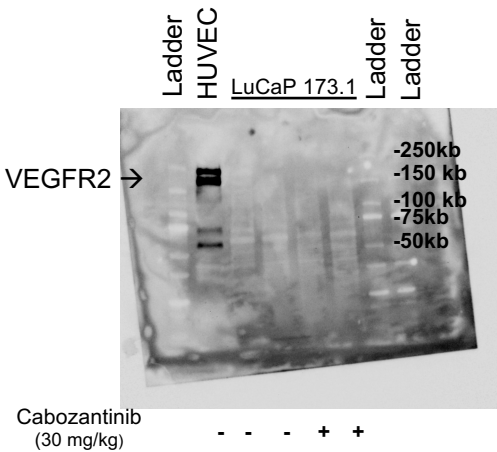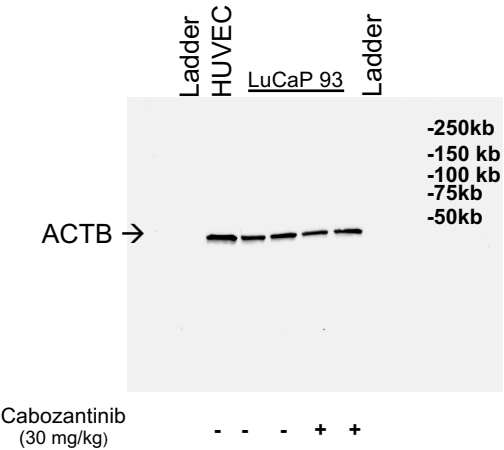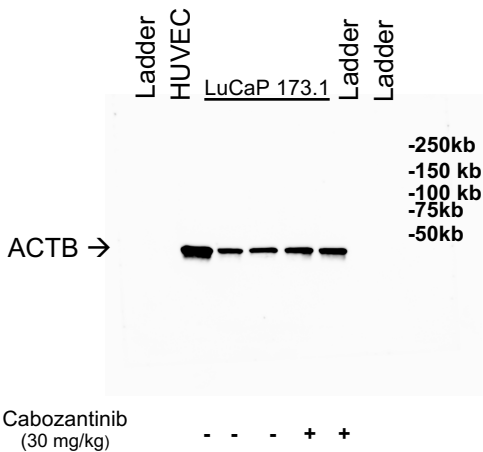

Supplement: S1 Raw images — (PDF) [file pone.0245602.s007.pdf]
